# Patency and maturation rates after forearm arteriovenous fistulas: systematic review with meta-analysis

**DOI:** 10.1007/s40620-025-02346-x

**Published:** 2025-07-29

**Authors:** Diogo Cristino, João Rocha Neves, Ryan Melo, Mario D’Oria, José Oliveira-Pinto

**Affiliations:** 1 Faculty of Medicine of the University of Porto, Porto, Portugal; 2 Department of Biomedicine, Unity of Anatomy, Faculty of Medicine of the University of Porto, Al. Prof. Hernâni Monteiro, 4200-319 Porto, Portugal; 3 RISE-Health, Departamento de Biomedicina-Unidade de Anatomia, Faculdade de Medicina, Universidade do Porto, Porto, Portugal; 4 Vascular Surgery Department, Hospital Santa Maria, Unidade Local de Saúde de Santa Maria, Faculdade de Medicina Universidade de Lisboa, Centro Cardiovascular da Universidade de Lisboa (CCUL@RISE), Lisbon, Portugal; 5 Division of Vascular and Endovascular Surgery, Department of Clinical Surgical and Health Sciences, University of Trieste, 34127 Trieste, Italy; 6 Cardiovascular R&D Centre-UnIC @RISE, Department of Surgery and Physiology, Faculty of Medicine of the University of Porto, Porto, Portugal; 7 Department of Angiology and Vascular Surgery, Unidade Local de Saúde de Trás-os-Montes e Alto Douro, Vila Real, Portugal

**Keywords:** Arteriovenous shunt, Surgical, Cimino-brescia fistula, Renal dialysis, Primary patency, Radiocephalic fistula

## Abstract

**Background:**

The growing burden of chronic kidney disease has significantly increased the demand for hemodialysis, with reliable vascular access being vital for treatment success. Although the radiocephalic arteriovenous fistula (AVF) remains the first option as a long term hemodialysis access, challenges related to aging patient population and prevalence of comorbidities may compromise maturation and durability. Therefore, this systematic review with meta-analysis aims to determine the patency and maturation rates of radiocephalic AVF, focusing on how different patient characteristics and surgical methodologies impact these rates.

**Materials and methods:**

Pubmed, Web of Science and Cochrane were systematically searched for studies assessing patency and maturation rates of radiocephalic AVF. Primary and secondary endpoints were pooled by random-effects meta-analysis, with sources of heterogeneity being explored by meta-regression.

**Results:**

Thirty-six cohort studies were selected, 15 of which were prospective. The meta analytical incidence of primary patency rates at 30 days, and at 1, 2, and 3 years after fistula creation was 84.18%, 68.43%, 59.86% and 55.10%, respectively. Additionally, the meta-analytical incidence of maturation failure, infection, aneurysm degeneration, early-thrombosis and reintervention rates was 24.7%, 3.9%, 3.6%, 6.3% and 29.4%, respectively. All the variables previously referred had high levels of heterogeneity associated. Furthermore, meta-regression analysis identified previous vascular access construction and latero-lateral anastomosis as factors associated with higher maturation failure rates.

**Conclusion:**

The significant heterogeneity observed in the endpoints evaluated highlight the complexity of radiocephalic AVF management. These findings underline the need for standardized clinical practices and further large-scale studies to identify factors influencing radiocephalic AVF outcomes.

**Graphical abstract:**

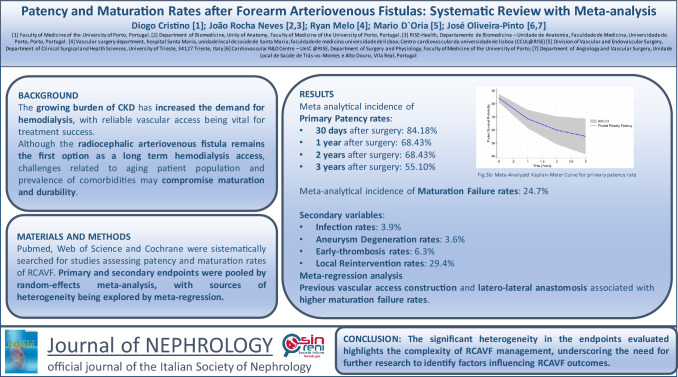

**Supplementary Information:**

The online version contains supplementary material available at 10.1007/s40620-025-02346-x.

## Introduction

The increased prevalence of chronic kidney disease (CKD) and the subsequent progression to kidney failure is associated with early mortality, decreased quality of life, and increased health-care expenditures [1].

Well-functioning arteriovenous fistula (AVF) is a critical requirement to initiate and maintain successful kidney replacement therapy [2]. According to the 2019 KDOQI Clinical Practice Guideline for Vascular Access, the ideal vascular access for hemodialysis should ensure reliable, complication-free delivery of the prescribed treatment while also being appropriately tailored to the individual needs of the patient [3].

The autogenous AVF is the preferred method of vascular access for hemodialysis due to better performance when compared to prosthetic grafts [4]. In 1966, Brescia, Cimino, Appel, and Hurlwith described a surgically created internal AVF at the wrist between the radial artery and the cephalic vein [5]. Since then, it has been generally accepted as the fistula with the longest patency and fewest complications [6]. Besides that, this type of radiocephalic AVF is usually easy to cannulate and allows to preserve venous patrimony proximally [7].

However, unlike the population in the 60’s, nowadays the mean age of the dialysis population is over 60 years old, and diabetes mellitus (DM) is present in about 50% of the population in some studies [8].

The aim of this study was to comprehensively assess the patency and maturation rates of radiocephalic AVF, with a particular focus on how different patient characteristics and surgical methodologies impact these rates. In order to fill the present gap, this review aimed to gather the most recent information concerning the incidence and predictors of patency and maturation after radiocephalic AVF creation.

## Materials and methods

### Study design

This systematic review was conducted in accordance with the Preferred Reporting Items for a Systematic Review and Meta-analysis (PRISMA) Statement and the AMSTAR-2 critical appraisal tool [9, 10]. Institutional review board ethical approval was not obtained due to the nature of this study. The review protocol has been registered at PROSPERO (reference: CRD42023491961).

### Selection criteria

Inclusion criteria consisted in all original studies performed in humans (except for systematic reviews, case reports and conference proceedings) in which the patency and maturation rates after radiocephalic arteriovenous fistula creation was assessed. No exclusion criteria based on the publication language or date were applied.

### Search strategy

A systematic search was performed in three databases—Pubmed, Web of Science and Cochrane—, on 31st December, 2024. The query is shown in Supplemental Table 1. Additionally, the references of the included primary studies and relevant available systematic reviews were screened to search for any further articles of possible interest.

**Table 1:** Procedural protocols implemented in the included studies

Author	AVF site	Anastomosis type	Anesthesia type	Preoperative assessment	Postoperative assessment
Wetzig et al.	Wrist	End-to-side	Local	N/A	Evaluation of audible bruit, palpable thrill and pulsation in the venous limb, and brachial arteriography
Wang et al.	Wrist	End-to-side	N/A	Clinical evaluation	Access flow monitoring during hemodialysis
Weale et al.	N/A	End-to-side	Local	Clinical evaluation, and duplex ultrasonography	N/A
Zeebregts et al.	Wrist	End-to-side	General or Regional block	Clinical evaluation (including Allen’s test), and duplex ultrasound	N/A
Prischl et al.	Wrist	End-to-side	Local	N/A	Clinical evaluation
Alm et al.	Forearm	End-to-side, side-to-side, side-to-end or end-to-end	Local or Regional block	N/A	N/A
Lindfors et al.	Wrist	Side-to-side	Local	N/A	N/A
Wong et al.	Wrist	End-to-side	Local	Clinical and ultrasound assessment of the cephalic vein, and radial artery	Non-invasive Doppler flow measurement and B-mode and color flow imaging of the cephalic vein, radial artery, and the anastomotic region
Lin et al.	N/A	Side-to-end	Local	N/A	Color duplex ultrasonography of the cephalic vein
Miller et al.	Forearm and upper arm	N/A	N/A	N/A	N/A
Golledge et al.	Wrist	Side-to-side	N/A	Demonstration of a normal radial pulse and distensible veins at the wrist	Clinical evaluation, and arteriogram
Huseynova et al.	Wrist	End-to-side	Local, general or regional block	Ultrasound vein mapping	Clinical evaluation, and ultrasound or fistulagram
Prasad et al.	Forearm	End-to-side	N/A	Doppler imaging of the vessels	N/A
Ramanathan et al.	Forearm	End-to-side	Local, general or regional block	Clinical evaluation (including Allen’s test), and vein assessment with a tourniquet or duplex scan	Clinical evaluation
Anil et al.	Wrist	End-to-side or side-to-side	Regional block	Clinical evaluation, and duplex ultrasound	Evaluation of audible bruit or palpable thrill in physical examination, and access flow monitoring during hemodialysis
Xu et al.	Wrist	End-to-side	Local	Clinical evaluation, and ultrasound vessel size measurement	Clinical evaluation and access flow monitoring during hemodialysis. Ultrasound or angiography if signs of dysfunction
Srivastava et al.	Wrist	End-to-side	N/A	Clinical evaluation (including Allen’s test), and duplex ultrasound	Clinical evaluation and access maximum flow monitoring during hemodialysis
Jemcov et al.	Forearm	End-to-side	N/A	Physical and color Doppler ultrasound assessment of blood vessels	Measurement of flow through the AVF, and cephalic vein distensibility
O’Banion et al.	Wrist	End-to-side or side-to-side	N/A	Clinical evaluation, and venography	Evaluation of audible bruit or palpable thrill in physical examination
Won et al.	Wrist	End-to-side	Local	N/A	Evaluation of audible bruit or palpable thrill in physical examination, and access flow measurements
Elsayed et al.	Forearm	N/A	N/A	Clinical evaluation (with modified Allen’s test) and ultrasound vessel assessment	Color Doppler ultrasound
Bhalodia et al.	Forearm and wrist	Side-to-side	N/A	Ultrasound vascular mapping	Clinical evaluation, and ultrasound
Mehigan et al.	Snuffbox	Side-to-side	Local	N/A	N/A
Wolowczyk et al.	Snuffbox	End-to-side	Local	Clinical evaluation and ultrasound vascular mapping	Clinical evaluation (palpable thrill)
Bonalumi et al.	Snuffbox	End-to-end	Local	N/A	Clinical evaluation
Twine et al.	Snuffbox	N/A	N/A	Arterial and venous duplex ultrasound	Clinical evaluation
Horimi et al.	Snuffbox	N/A	N/A	Clinical evaluation, and ultrasound	Clinical evaluation
Cassioumis et al.	Wrist	End-to-side, side-to-side or end-to-end	N/A	N/A	N/A
Reilly et al.	Wrist	End-to-side, side-to-side	Local	N/A	Clinical evaluation, and vessel size measurement
Thompson et al.	Wrist	Side-to-side or end-to-end	Regional block	N/A	Clinical evaluation, arteriogram, and cardiac output measurement
Kherlakian et al.	Wrist	End-to-side, side-to-side or end-to-end	Local	Clinical, physical, radiologic, and hemodynamic evaluation	Clinical and physical evaluation
Bender et al	Wrist	Side-to-end	N/A	N/A	Clinical evaluation
Burt et al.	Wrist	End-to-side	N/A	N/A	Clinical evaluation
Lok et al.	N/A	N/A	N/A	N/A	Clinical evaluation, and Access flow monitoring
Mishra et al.	Wrist	End-to-side	Local	Clinical evaluation, and pre-operative Doppler ultrasound	Clinical evaluation, and Doppler ultrasound
Elshikhawoda et al.	Snuffbox and Wrist	End-to-side	Local	N/A	Clinical evaluation, and Doppler ultrasound

*AVF* arteriovenous fistula, *N/A* not available

### Study selection and data extraction

After removing duplicates, two authors (DC and JRN) independently participated in study selection; any disagreement was solved by the intervention of a third author (JOP). First, studies were selected by title and abstract, and the remaining ones were eligible for full-text assessment. Efforts were made to contact the authors to obtain the full texts that were not publicly available. The selected studies were carefully revised to avoid repeated populations.

Data from included studies were independently extracted by two authors (DC and JRN), using a purposely-built form on the year of publication, country, recruitment center, study design, recruitment time, number of participants undergoing radiocephalic fistula placement, participants’ age and gender distribution, frequency of cardiovascular comorbidities and patency. WebPlotDigitaliser software was utilized to extract data regarding primary and secondary patency rates.

### Assessment of study quality

Concerning qualitative assessment, the National Heart, Lung, and Blood Institute (NHLBI) Study Quality Assessment Tool was used for observational cohort and cross-sectional studies (2013). This assessment was independently performed by two authors (JRN and DC), and when disagreements occurred, decisions were made by mutual consensus after a third-party review (JOP).

### Quantitative synthesis

A random-effects meta-analysis (by the restricted maximum likelihood method) was used to calculate the meta-analytical pooled incidence of access failure among participants. Heterogeneity was assessed using the Q-Cochran *p* value, and the *I*^2^ statistic—a *p* value < 0.10 and an *I*^2^ ≥ 50% were considered to represent substantial heterogeneity. Sources of heterogeneity were assessed by univariable meta-regression models. Only covariates that were reported in more than five studies underwent meta-regression. Assessed covariates included the participants’ mean age, percentage of male participants, percentage of patients with arterial hypertension, diabetes, location of the fistula (snuffbox, wrist or forearm), presence of previous vascular access, arterial and venous diameter and type of anastomosis. Subgroup analyses were also performed with separate analyses of studies published before and after the year 2000.

All statistical analyses were performed using OpenMeta[Analyst] version 0.24.1 (MetaMorph Inc., 2023).

## Results

### Search results

**Fig.1:**
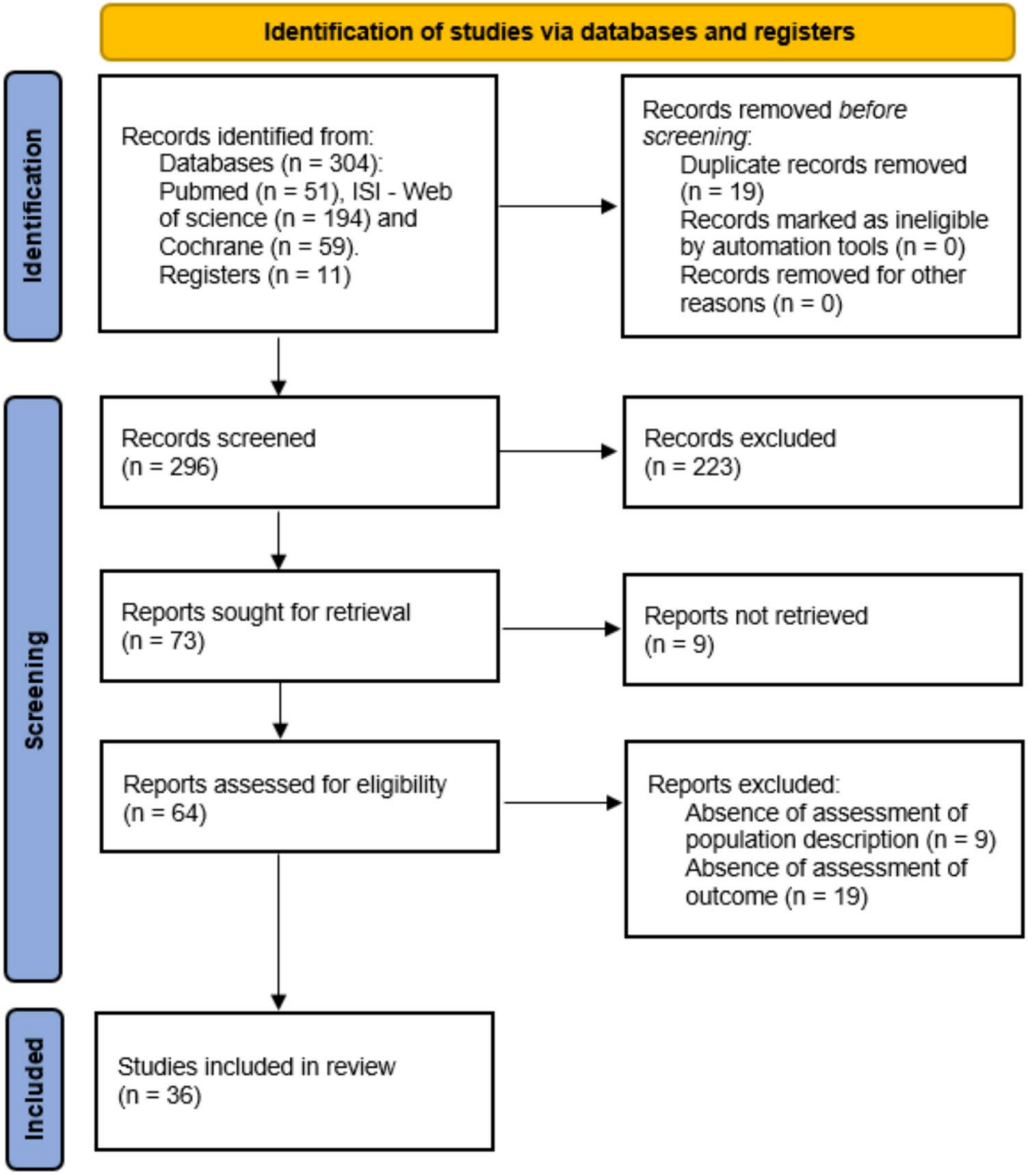
PRISMA 2020 flow diagram for new systematic reviews which included searches of databases and registers only. From: Page MJ, McKenzie JE, Bossuyt PM, Boutron I, Hoffmann TC, Mulrow CD, et al. The PRISMA 2020 statement: an updated guideline for reporting systematic reviews. BMJ 2021;372: n71. 10.1136/bmj.n71. For more information, visit: http://www.prisma-statement.org/

After the database search and duplicate exclusion, a total of 296 studies were screened. Upon selection by title and abstract, 223 studies were excluded. 73 studies were eligible for full-text assessment, and, during this process, 28 studies were excluded, and 9 were not retrieved (Fig. 1). Comprehensive reasons for exclusion upon full-text assessment were: absence of assessment of population description (*N* = 9), absence of assessment of outcome (*N* = 19), and the inability to access the full text despite multiple attempts to contact the respective author (*N* = 9). Thus, a total of 36 published articles were included in this systematic review.

### Description of studies

All 36 studies included in this systematic review were observational cohorts, with 15 of them being prospective [11–25] and 21 of them being retrospective [2, 4–8, 26–40]. The included publications were carried out in 18 different countries within 4 continents—16 from Europe [2, 5–7, 11, 19, 21–24, 31, 34–36, 39, 40], 9 from North America [4, 8, 15, 17, 26, 27, 33, 38, 41], 9 from Asia [12–14, 16, 18, 20, 25, 28, 29], and 2 from Oceania [30, 32]. A total of 4584 patients were assessed, with a minimum of 18 and a maximum of 500 patients per study. Mean age of the participants was 52 years old, and 65% were male (*N* = 2498/3818). Demographics and comorbidities of the populations included in the studies were gathered and are available in Supplemental Table 2. Procedural protocols carried out in each of the included studies are detailed in Table 1.

### Outcome definition

In this review, several definitions of maturation failure were identified, varying from a radiocephalic arteriovenous fistula that was left to mature for 6 weeks, 6 months or until the end of follow-up period, during which it was evaluated and found to be unsuitable for adequate hemodialysis. The 6-week and 6-month marks were selected because they were the most frequently chosen time intervals in the studies reviewed. Note that maturation failure was defined as an AV access that, despite radiologic or surgical intervention (endovascular or open procedures), could not be used successfully for hemodialysis by the end of the follow-up period. However, most studies either omitted a precise definition of maturation failure or used heterogeneous criteria, underscoring the need for standardized definitions in accordance with the 2019 KDOQI Clinical Practice Guideline for Vascular Access across studies.

Primary patency rates (intervention-free access survival) were defined as the number of functioning AVFs at 30 days, and 1, 2 and 3 years after AVF creation in which no interventions designed to maintain or re-establish patency were performed, divided by the total number of AVFs created. Secondary patency rates were defined as the number of functioning AVFs at 1, and 2 years after creation, including all AVFs which underwent interventions (surgical or endovascular interventions) designed to re-establish functionality in a thrombosed access, divided by the total number of AVFs created.

Regarding the definition of aneurysm degeneration, both true vascular access aneurysms (involving all layers of the vessel) and false aneurysms were included. Additionally, regarding re-intervention rates, re-intervention is defined as any additional medical or surgical procedure performed after the initial creation of an AVF to address complications such as stenosis or thrombosis. Common re-intervention techniques include balloon angioplasty, stent deployment, mechanical thrombectomy, pharmacologic thrombolysis, and surgical revisions.

Regarding infections occurring during the perioperative period, although we initially intended to use the definition of the Centers for Disease Control (CDC) for vascular access-related infections, most of the definitions reported in the included studies could not be included in this category. Therefore, infection rates were defined in a broader sense, including access site infections, access-related bloodstream infections, and infections not otherwise specified.

Lastly, in the analysis of early-thrombosis rates at 24 h after surgery, only complete occlusive access thrombosis was considered. This is reflected in the absence of a bruit or thrill, determined by using auscultation and palpation, throughout systole and diastole proximally to the arteriovenous anastomosis [42].

### Main findings and meta-analysis

#### Primary variables

##### Maturation failure rates

**Fig. 2:**
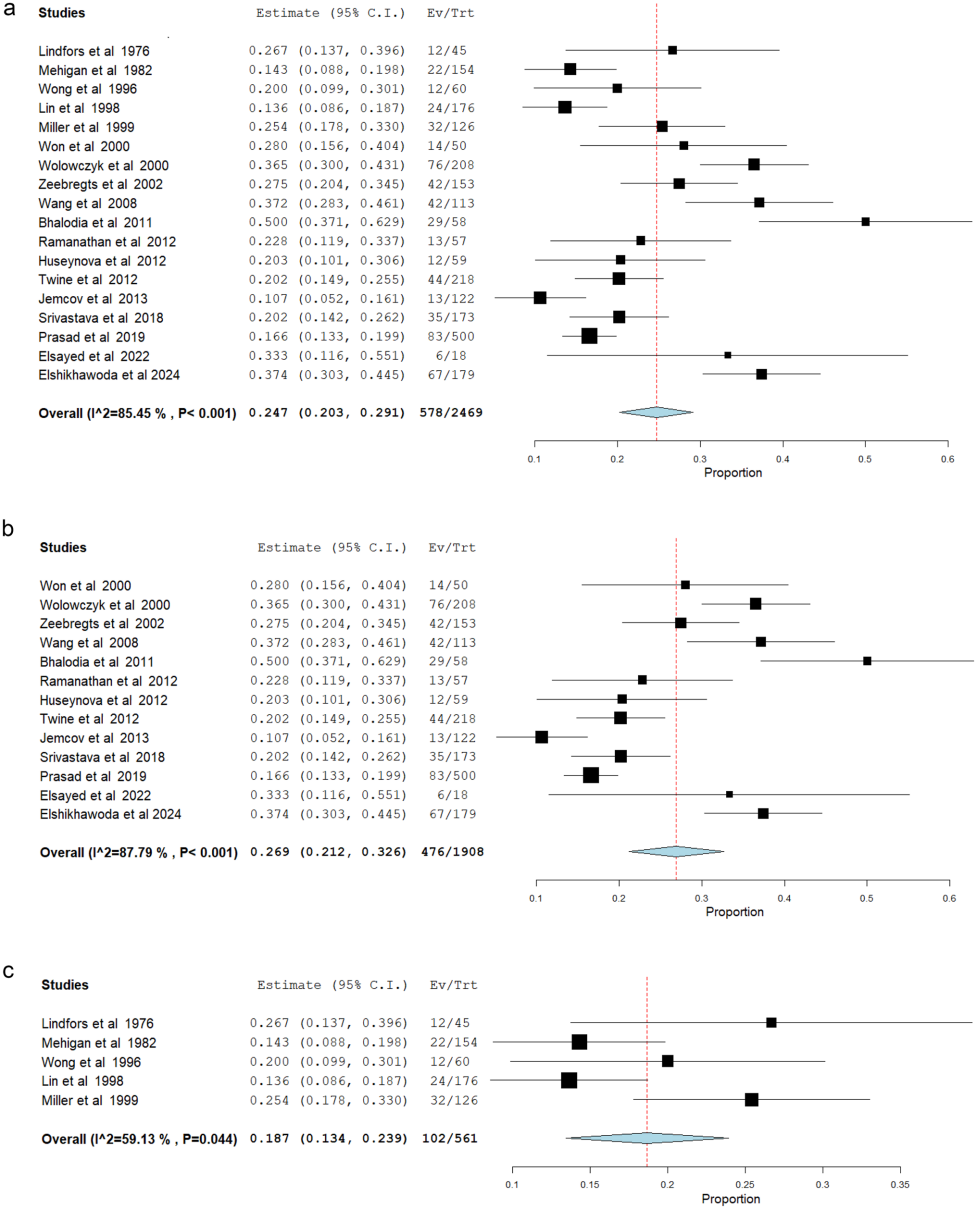

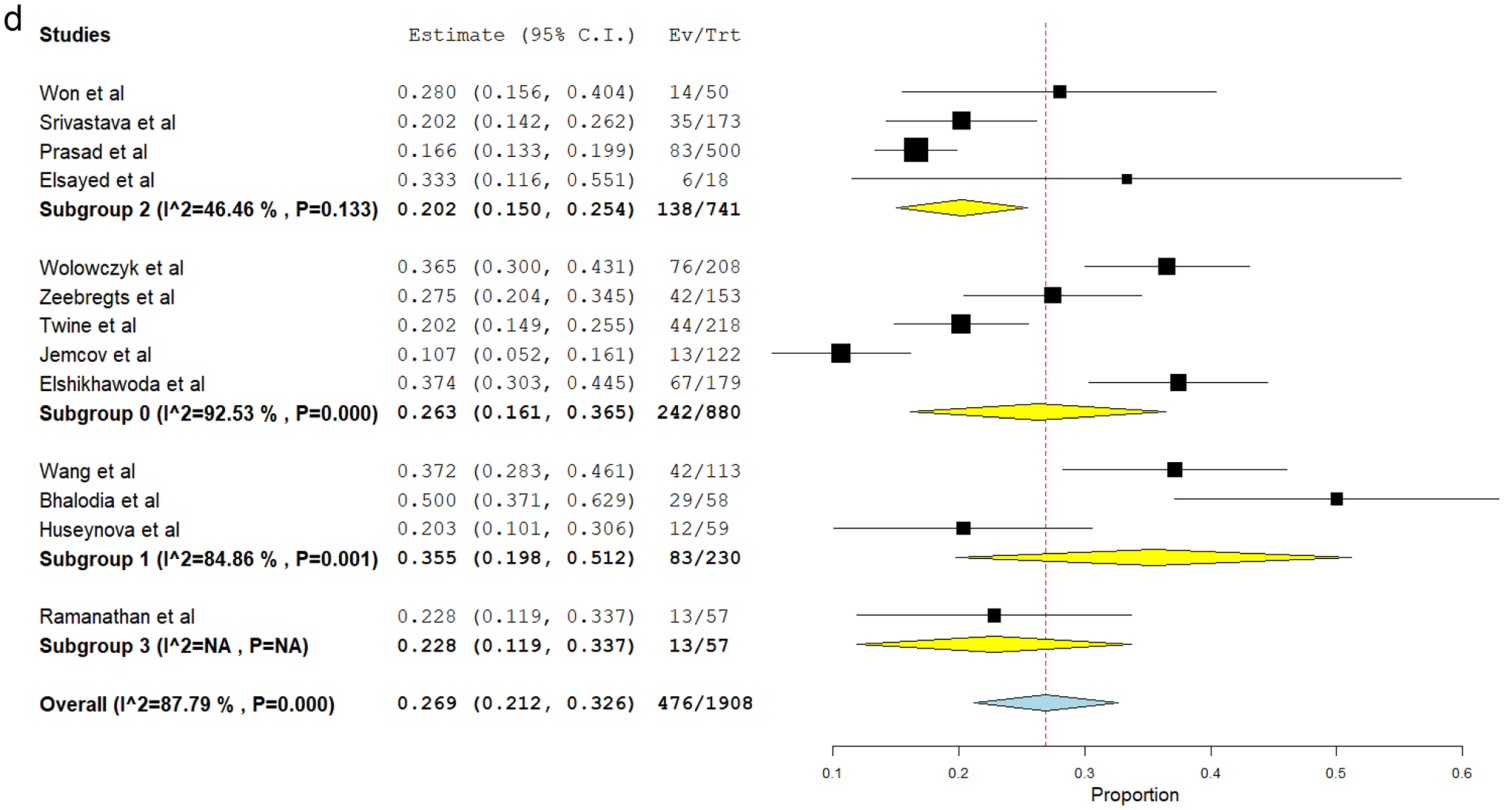
**a** Meta-analysis of maturation failure rates at the end of the follow-up period (overall). **b** Meta-analysis of maturation failure rates at the end of the follow-up period (studies after the year 2000). **c** Meta-analysis of maturation failure rates at the end of the follow-up period (studies before the year 2000). **d** Meta-analysis of maturation failure rates at the end of the follow-up period subdivided by the continent of the study center

Eighteen studies [5, 7, 12, 14–19, 22, 24, 26, 28, 30, 33, 37, 39, 40] (*N* = 2469) reported the incidence of maturation failure after radiocephalic AVF placement, at the end of the follow up period. This incidence ranged from 10.7% [22] to 50.0% [33]. The meta-analytical incidence was 24.7% [95% CI 20.3–29.1%]. However, severe heterogeneity was found (*I*^2^ = 85.45%; Q-Cochran *p* value < 0.001) (Fig. 2a). Considering only the patients who underwent radiocephalic AVF creation after the year 2000 [7, 12, 16–18, 22, 24, 26, 28, 30, 33, 39, 40] (*N* = 1908), the meta-analytical incidence of maturation failure was 26.9% [95% CI 21.2–32.6%]. This subpopulation also showed severe heterogeneity (*I*^2^% = 87.79%; Q-Cochran *p* value < 0.001) (Fig. 2b). In comparison, regarding patients who underwent radiocephalic AVF before the year 2000 [5, 14, 15, 19, 37] (*N* = 561), the meta-analytical incidence of maturation failure was 18.7% [95% CI 13.4–23.9%]. While lower than the subpopulation that underwent radiocephalic AVF after the year 2000, high levels of heterogeneity were also found in this subpopulation (*I*^2^% = 59.13%; Q-Cochran *p* value = 0.044) (Fig. 2c). To better address the high level of heterogeneity previously observed, the thirteen studies published after the year 2000 were separated into 4 subgroups, according to the continent of origin. Subgroup 0 contains the studies from Europe [7, 22, 24, 39, 40] (*N* = 880), and the meta-analytical incidence of maturation failure was 26.3% [95% CI 16.1–36.5%], with severe heterogeneity (*I*^2^% = 92.53%; Q-Cochran *p* value < 0.001). Subgroup 1 contains the studies from North America [17, 26, 33] (*N* = 230), and the meta-analytical incidence of maturation failure was 33.5% [95% CI 19.8–51.2%], with high levels of heterogeneity (*I*^2^% = 84.86%; Q-Cochran *p* value = 0.001). Subgroup 2 contains the studies from Asia [12, 16, 18, 28] (*N* = 741), and the meta-analytical incidence of maturation failure was 20.2% [95% CI 15.0–25.4%], with lower levels of heterogeneity compared to the other subgroups (*I*^2^% = 46.46; Q-Cochran *p* value = 0.133). Subgroup 3 contains the only study from Oceania [30] (*N* = 57), and the incidence of maturation failure was 22.8% [95% CI 11.9–33.7%] (Fig. 2d). In further analysis, maturation failure rates were subdivided by different follow-up times. Despite these efforts, follow-up times regarding maturation failure rates varied widely, with only three studies reporting maturation failure rates at 6 weeks [7, 24, 39] and three studies reporting maturation failure rates at 6 months [15, 33, 37]. Regarding the 6-week mark, maturation failure rates were 5.96% [24], 27.45% [7], and 36.54% [39]. Furthermore, maturation failure rates at the 6-month mark were 14.29% [37], 50% [33], and 66% [15].

##### Primary patency rates

**Fig. 3:**
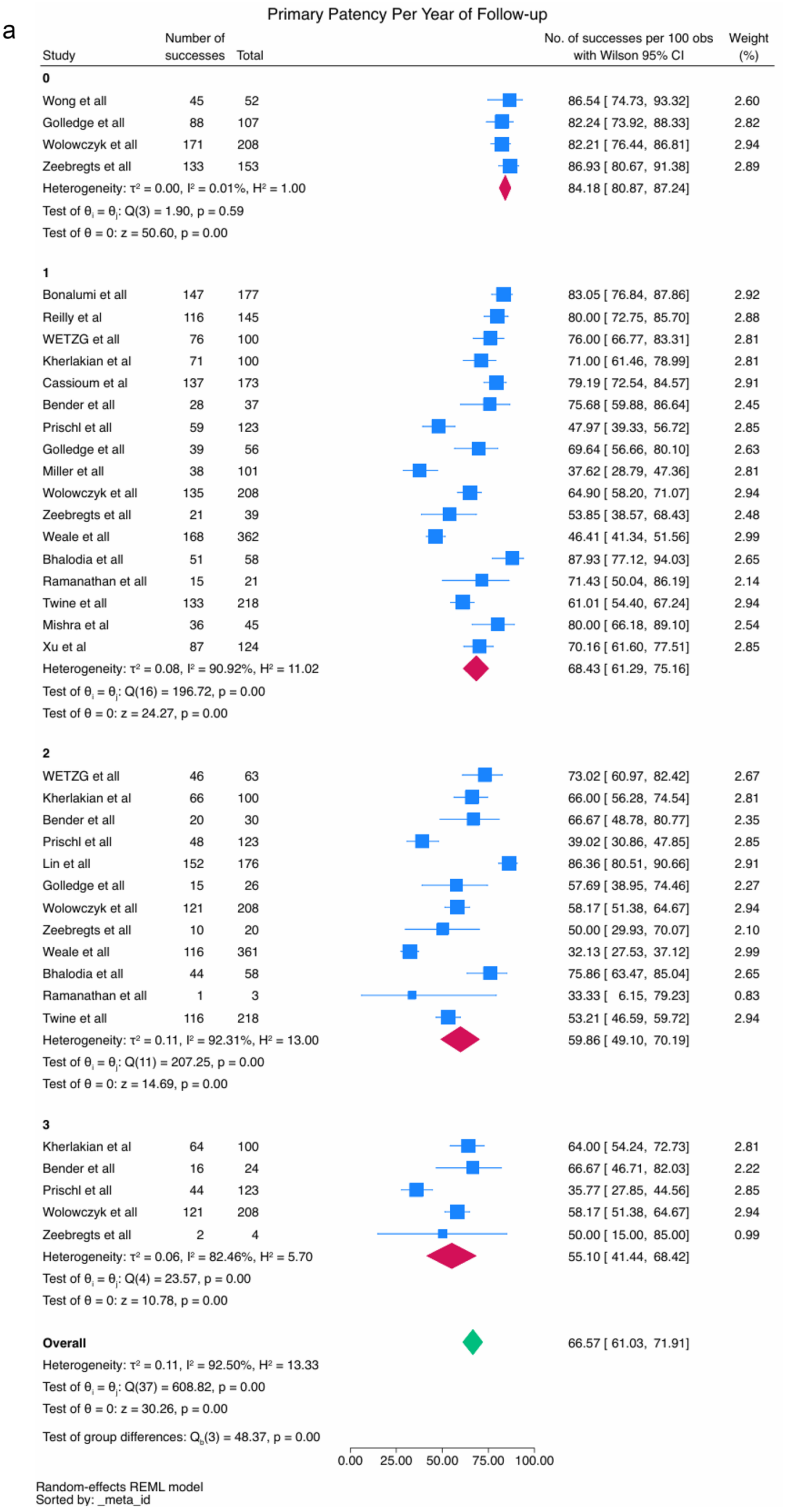

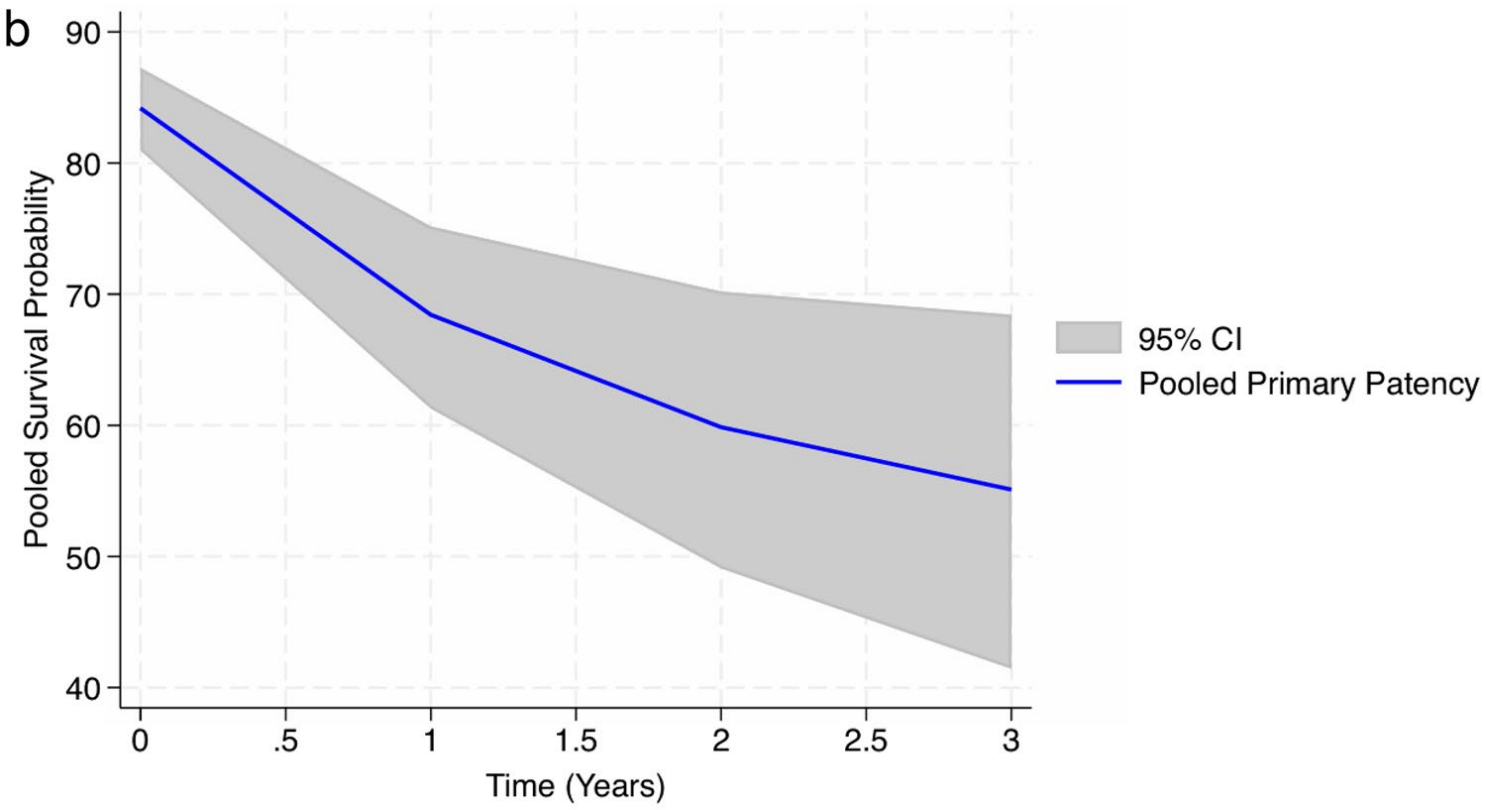
**a** Meta-analysis of primary patency rates at 30 days (subgroup 0), and at 1 (subgroup 1), 2 (subgroup 2) and 3 (subgroup 3) years. **b** Meta-Analyzed Kaplan–Meier curve for primary patency rates

Data on primary patency rates at 30 days, and 1, 2 and 3 years after radiocephalic AVF creation were collected. Four studies [7, 19, 21, 39] (*N* = 502) reported primary patency rates at 30 days after AVF creation, with values ranging from 82.21% [39] to 86.93% [7]. The meta-analytical incidence was 84.18% [95% CI 80.87–87.24%], with very low heterogeneity levels observed (*I*^2^% = 0.01%); Q-Cochran *p* value = 0.59). Twenty-two studies [2, 4, 6–8, 13, 15, 20, 21, 23–26, 28, 30–32, 34–36, 39] (*N* = 3266) reported primary patency rates at 1 year after radiocephalic AVF creation. These rates ranged from 38% [15] to 96% [13]. The meta-analytical incidence was 68.43% [95% CI 61.29–75.16%], with high heterogeneity levels observed (I^2^% = 90.92%); Q-Cochran *p* value = 0.00). Regarding the 2-year mark, fifteen studies [2, 4, 6–8, 13, 14, 21, 24, 30–33, 35, 39] (*N* = 2120) reported primary patency rates at this timeline, with rates ranging from 32.1% [31] to 89% [13, 14]. The meta-analytical incidence was 59.86% [95% CI 49.10–70.19%], with high heterogeneity levels observed (*I*^2^% = 92.31%); Q-Cochran *p* value = 0.00). Lastly, regarding the 3-year mark, seven studies [2, 4, 6–8, 13, 39] (*N* = 1013) reported primary patency rates at this timeline. These rates ranged from 36% [2] to 87% [13]. The meta-analytical incidence was 55.10% [95% CI 41.44–68.42%], with high heterogeneity levels observed (*I*^2^% = 82.46%); Q-Cochran *p* value = 0.00). (Fig. 3a and 3b).

##### Secondary patency rates

Additionally, data regarding secondary patency rates at 1, and 2 years after radiocephalic AVF creation were also collected. Six studies [20, 21, 28, 30, 31, 35] (*N* = 1202) reported primary patency rates at 1 year after radiocephalic AVF creation. These rates ranged from 46.4% [31] to 87.6% [30]. Regarding the 2-year mark, four studies [21, 30, 31, 35] (*N* = 578) reported secondary patency rates at this timeline, with rates rang ing from 32.1% [31] to 83.9% [30]. No study on this systematic review protocol reported secondary patency rates at 30 days or 3 years after radiocephalic AVF creation.

#### Secondary outcomes

##### Aneurysm degeneration

**Fig. 4:**
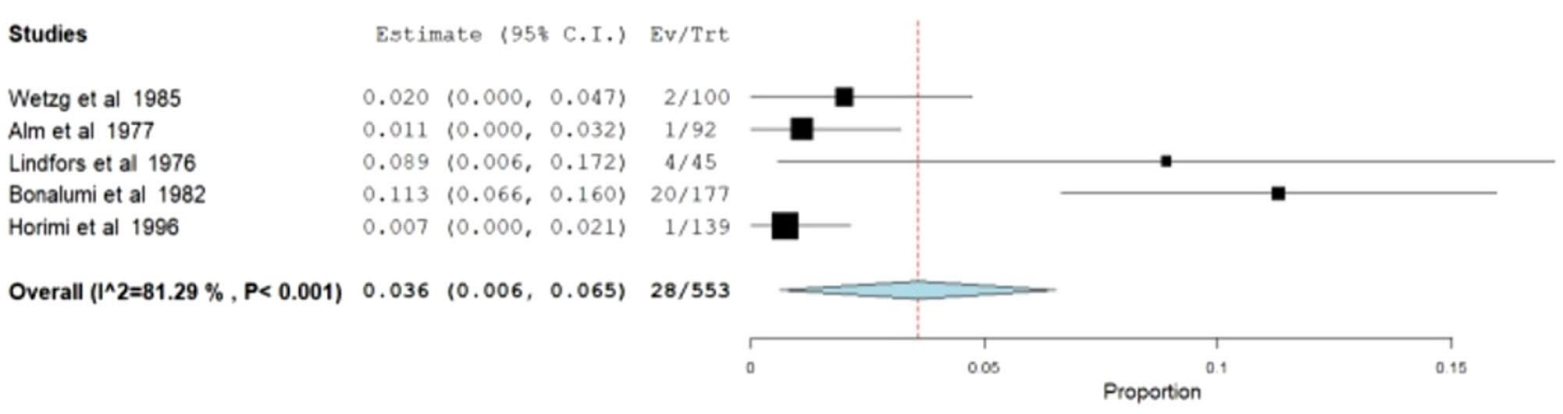
Meta-analysis of aneurysm degeneration rates

Incidence of aneurysm degeneration at the anastomosis site was also analyzed. Five studies reported this metric [5, 11, 13, 32, 34] (*N* = 553) with rates ranging from 0.7% [13] to 11.3% [34]. The meta-analytical incidence was 3.6% [95% CI 0.6–6.5%]. This metric was also associated with high values of heterogeneity (*I*^2^ = 81.286%; Q-Cochran *p* value < 0.001). (Fig. 4).

##### Need for reintervention

**Fig. 5:**
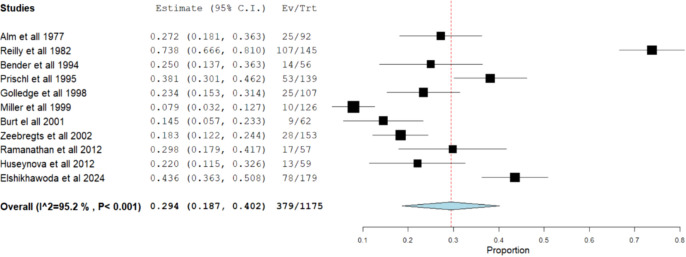
Meta-analysis of local reintervention rates

A total of eleven studies [2, 6, 7, 11, 15, 21, 23, 26, 30, 35, 40] (*N* = 1175) were included in the analysis of local reintervention rates after forearm AVF creation. The local reintervention incidence ranged from 7.9% [15] to 73.8% [23] and the meta-analytical incidence was 29.4% [95% CI 18.7–40.2%]. However, severe heterogeneity was found (*I*^2^ = 95.2%; Q-Cochran *p* value < 0.001). (Fig. 5).

##### Early-thrombosis and infection rates

**Fig. 6:**
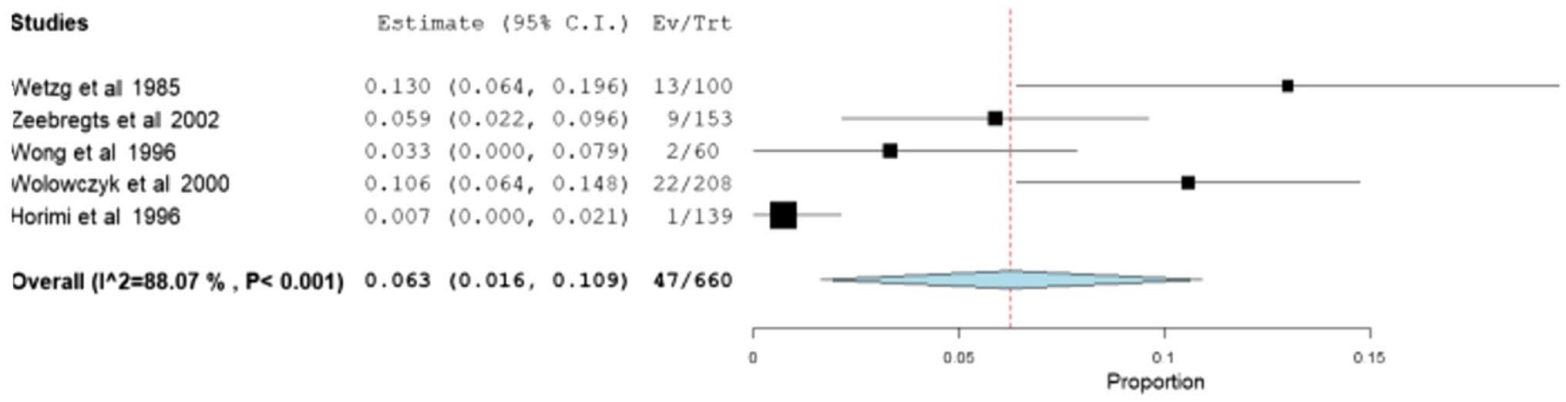
Meta-analysis of early-thrombosis rates

**Fig. 7:**
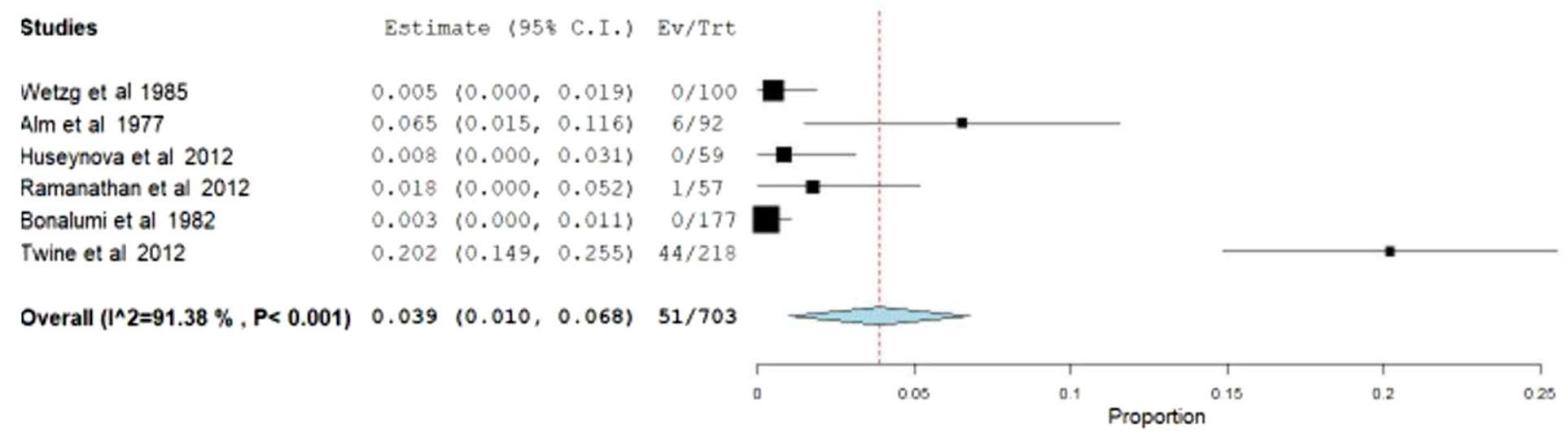
Meta-analysis of infection rates

Additionally, other secondary variables like early-thrombosis and infection rates were also evaluated. The meta-analytical incidence of early-thrombosis at 24 h after surgery was 6.3% [95% CI 1.6–10.9%], with severe heterogeneity associated (*I*^2^ = 88.07%; Q-Cochran *p* value < 0.001) (Fig. 6). Regarding infection rates during the perioperative period, the meta-analytical incidence was 3.9% [95% CI 1–6.8%], with also high levels of heterogeneity associated (*I*^2^ = 91.38%; Q-Cochran *p* value < 0.001) (Fig. 7).

Values regarding primary and secondary variables in each of the included studies are shown in Supplemental Table 3 and 4.

### Univariable meta-regression

**Fig. 8:**
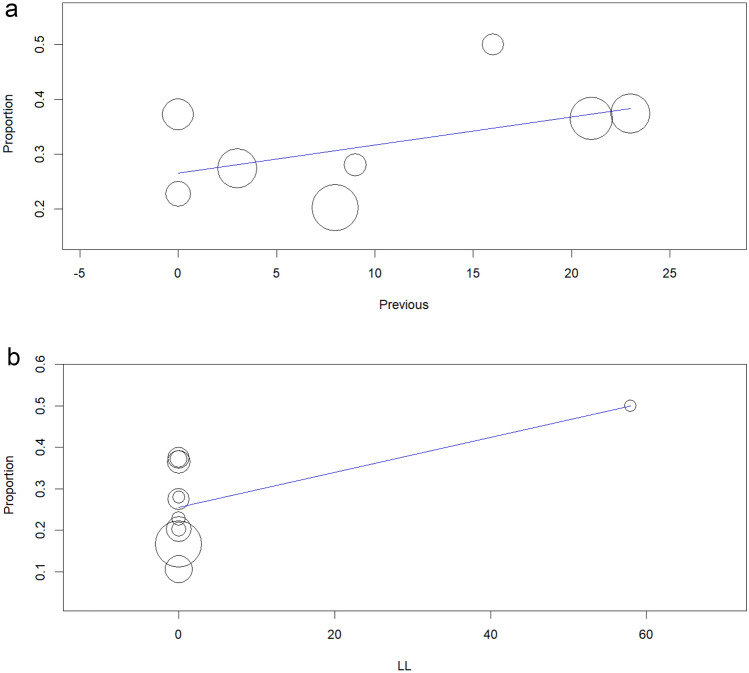
**a** Meta-regression analysis of the association between previous vascular access and maturation failure. **b** Meta-regression analysis of the association between latero-lateral anastomosis and maturation failure

Lastly, to better explore potential sources of heterogeneity and identify factors associated with radiocephalic fistula maturation failure, we conducted a formal meta-regression analysis. The meta-regression analysis, which included 8 studies, indicated that previous vascular access construction was associated with a tendency for radiocephalic fistula maturation failure (*β* = 0.5% 95% CI [− 0.1, 1.1]; *p* = 0.078) (Fig. 8a). Furthermore, meta-regression analysis including 11 studies indicated that the construction of a latero-lateral anastomosis was significantly associated with radiocephalic fistula maturation failure (*β* = 0.4% 95% CI [0.1, 0.8]; *p* = 0.023), with a positive coefficient suggesting an increased risk of failure (Fig. 8b). Further meta-regression analyses were conducted to examine the potential influence of other variables on radiocephalic AVF maturation failure. Notably, no significant associations were found between maturation failure and the percentage of male participants, prevalence of diabetes mellitus or hypertension, vein or artery diameter, or fistula location (wrist, forearm or snuffbox).

Additionally, meta-regression analysis revealed that early-thrombosis (within the first 24 h after surgery), irrespective of the specific definitions used across studies, showed no significant associations with evaluated covariates, indicating no consistent predictors were identified in this timeframe.

The odds ratio, confidence interval and p-value for each covariate analyzed in the meta-regression of maturation failure and early-thrombosis rates are shown in Supplemental Tables 5 and 6.

### Study quality

**Fig. 9:**
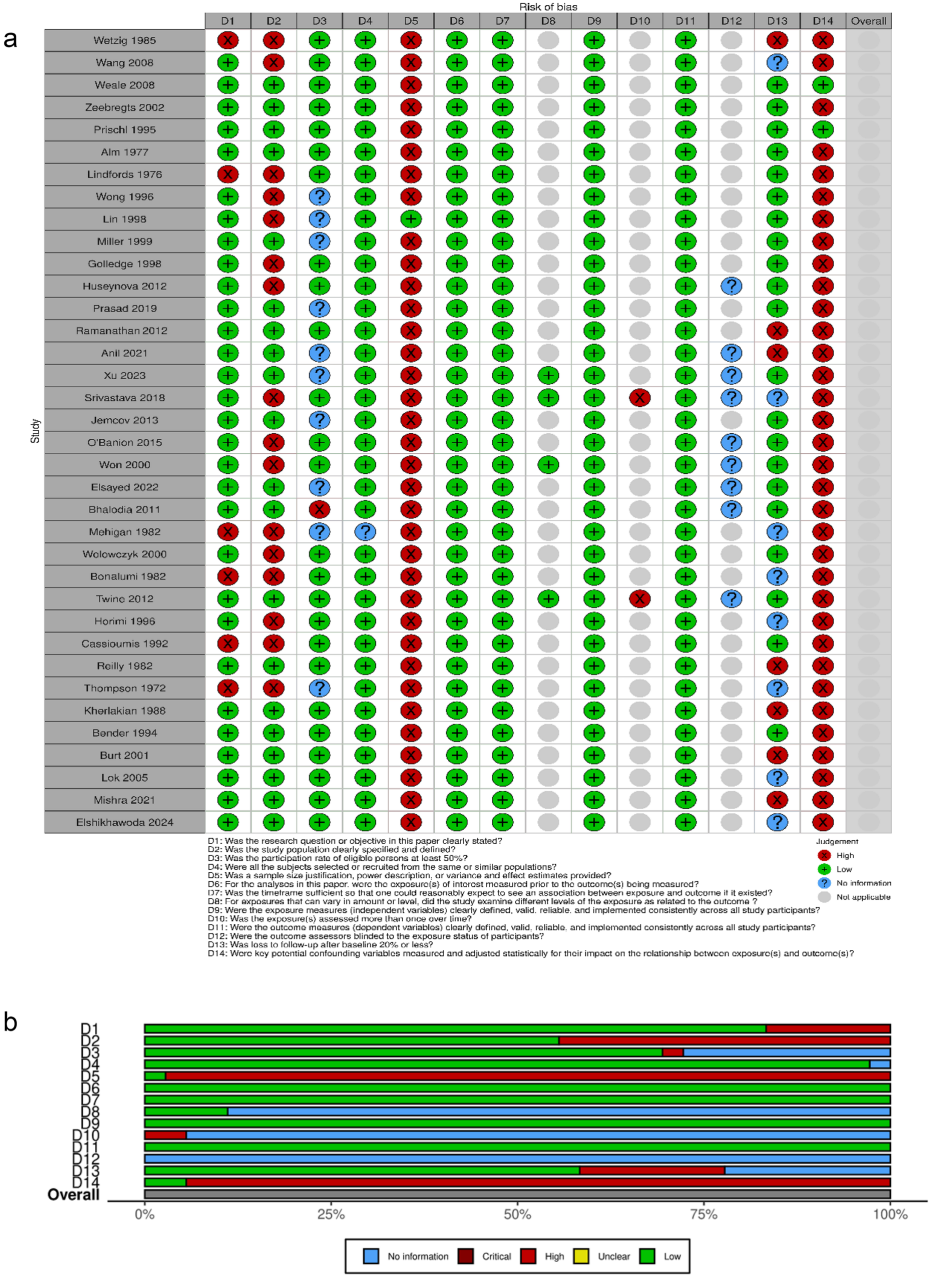
**a** Risk of bias of all the included studies in the systematic review, displayed by article. **b** Risk of bias of all the included studies, displayed by item

Risk of bias of the selected articles is displayed in Fig. 9a, b. The risk of bias for each observational cohort is individually displayed in Fig. 9a. The overall judgment per evaluated item regarding observational cohorts is shown in Fig. 9b.

All observational cohorts had an overall high risk of bias. The items most frequently associated with a high risk of bias amongst observational cohorts included sample size justification, power description, key potential confounding variables measurement, population definition, and loss to follow-up above 20%, in this order.

## Discussion

This systematic review with meta-analysis provides a comprehensive assessment of the outcomes associated with radiocephalic arteriovenous fistula creation, focusing primarily on patency and maturation rates during the follow-up period.

Meta-analytical incidence of maturation failure rates was 24.7%, with significant heterogeneity among studies. Subgroup analysis of this variable suggests slightly higher maturation failure rates in patients who underwent radiocephalic AVF creation after the year 2000. However, this difference was not statistically significant. Despite this stratified analysis, high levels of heterogeneity were still found in both subgroups, especially in the studies dated after the year 2000. Several factors may have influenced the differences observed between these two time frames. Over time, the reporting profile of the surgical candidates has improved dramatically. Also, compared to the population that underwent vascular access in the 1980s and the early 1990s, current hemodialysis patients are generally older, have higher rates of diabetes and cardiovascular comorbidities [43], and increasing risk of fistula failure [14]. Additionally, in older studies, maturation failure was mostly defined by clinical criteria (inability to puncture or dialytic insufficiency), while more recent studies mainly use ultrasonography. This factor may also contribute to the higher maturation failure rates detected in more recent studies, with Robin et al. [44] reporting that 19% of fistulas that were defined as mature by clinical criteria (easily palpable thrill and at least 10 cm of vein suitable for puncture) were deemed not mature by ultrasound evaluation.

Lastly, regarding the high variability in maturation failure rates in the 6-week and 6-month timeframes, a careful review of population demographics and procedural factors described in the six studies included was conducted. Regarding population demographics, the three studies included in each timeframe failed to consistently report variables like mean age, and percentage of patients with hypertension or diabetes mellitus. Additionally, the choice of anastomosis type was similar within each of the subgroups, rendering it unlikely to be the source of the variability observed. In contrast, there was a lack of standardization in the criteria used to define fistula maturation. Within the six studies reviewed, only four studies [7, 15, 33, 39] (two in each subgroup) clarified how fistula maturation was evaluated. Within these four studies, three [7, 15, 39] defined fistula maturation as a fistula that developed sufficiently to be used for hemodialysis, with Miller et al. [15] defining a minimum blood flow of 350 mL/min through the artificial kidney to consider a fistula mature. In contrast, Bhalodia et al. [33] defined fistula maturation more loosely, considering a fistula mature if it developed sufficiently to be used for hemodialysis or if ultrasound criteria were met. This factor could explain the lower maturation failure rate at 6 months observed in Bhalodia et al. (50%) when compared to Miller et al. (66%).

The other primary endpoints analyzed in this study were primary and secondary patency rates. Upon closer examination, it became evident that these values exhibited significant variability. Many of the included studies did not define patency rates consistently, with some studies including and others excluding primary failure from primary patency definition. Additionally, similarly to maturation rates, the heterogeneity observed can also be explained by different patient characteristics in the population, such as gender, incidence of DM, as well as study recruitment date. As mentioned earlier, more recent cohorts comprise an older demographic, with higher incidences of diabetes and cardiovascular comorbidities [43]. Since these factors have previously been linked with poor vessel quality [14], the wide variability in patency rates observed can also be attributed to this progressive change in patient characteristics.

Lastly, secondary outcomes such as aneurysm degeneration, local reintervention rates, infection and early-thrombosis rates were also evaluated. Infection and aneurysm degeneration rates were generally low, with meta-analytical incidences of 3.9% and 3.6%, respectively. In comparison, early-thrombosis and local reintervention rates were higher, with meta-analytical incidences of 6.3% and 29.4%. Regardless of the metric being studied, severe heterogeneity was consistently observed, underscoring the high complexity and variability in radiocephalic AVF outcomes across the various clinical settings evaluated.

Regarding local reintervention rates following radiocephalic AVF creation, contributing factors to the high heterogeneity among the included studies were carefully evaluated. The most frequent reintervention techniques performed were surgical thrombectomy (28.8%) and angioplasty (24.8%), with only Zeebregts et al. [7] providing data regarding the timing of reintervention: 10.7% of local reinterventions occurred on the same day as radiocephalic AVF creation, while the remainder were performed at a median of 18 months post-surgery. It is important to note that the type of procedure conducted in each study varied significantly. Although the overall proportion of angioplasties was 24.8%, this value reached as high as 84% [26] and 76% [30] in two individual studies. This asymmetrical distribution of interventions across studies, combined with the lack of reporting on the timing of these procedures, likely contributed to the substantial heterogeneity observed in this meta-analysis.

Lastly, the meta-analytical incidence of early-thrombosis, evaluated within the first 24 h post-surgery, was 6.3%, based on data from five studies. This metric also presented high levels of heterogeneity. Despite being noteworthy, thrombosis occurring so early after radiocephalic AVF creation is likely related to technical errors during the procedure or inadequate preoperative planning, with poor vessel quality assessment and selection. Therefore, even though this metric provides important insights, early thrombosis is largely preventable through correct surgical technique and preoperative planning.

Lastly, regarding the meta-regression findings, valuable insights into factors associated with radiocephalic AVF maturation failure were found. Firstly, although not statistically significant, previous vascular access construction was associated with a trend towards higher maturation failure rates. This finding is in line with recent literature, with Gibson et al. [45] stating that patients with previous vascular access placement had an 81% higher risk of primary access failure. Despite this, the borderline significance of the findings in this study warrants the need for further investigation to clarify this relationship. Secondly, regarding the type of anastomosis selected, the construction of a latero-lateral anastomosis was significantly associated with an increased risk of radiocephalic fistula maturation failure. Wedgewood et al [46] reported that latero-lateral fistulas can be associated with reduced total blood flow in the venous segments, leading to worsened tissue perfusion, and increased blood stasis, resulting in lower maturation rates. These findings are particularly important as they provide evidence of the impact of the surgical technique choice on fistula outcomes.

This study faced many limitations worth noting. First, few articles were eligible for this systematic review, and the majority of those that were had a small sample size without sample justifications and power descriptions, which led to low precision of obtained results. Moreover, there was severe heterogeneity amongst studies regarding most baseline patient characteristics, study designs, and methodology. In fact, differences between primary studies were so extensive that, in meta-regression, it was not possible to consider variables that could possibly account for most heterogeneity. Studies conducted before the year 2000 asymmetrically failed to report patient comorbidities, compared to more recent ones, rendering it impossible to address this limitation through meta-regression analysis. Few other short- and long-term outcomes were assessed.

## Conclusion

This systematic review with meta-analysis highlights the substantial variability in outcomes following radiocephalic arteriovenous fistula creation, particularly in maturation rates. The observed heterogeneity underscores the challenges in standardizing care across clinical settings and patient populations.

While the study identifies trends such as higher maturation failure in recent cohorts, the conclusions are limited by the inconsistent methodologies and small sample sizes of the included studies. To address these gaps, future research should focus on large-scale, well-designed studies aimed at standardizing protocols and identifying key factors influencing radiocephalic AVF success. This will be crucial for improving patient outcomes and optimizing care strategies.

## Supplementary Information

Below is the link to the electronic supplementary material.

Supplementary file1 (DOCX 35 KB)

## Declarations

### Data availability

No primary data were used.
