# Supplementary material for: Patency and maturation rates after forearm arteriovenous fistulas: systematic review with meta-analysis
Source: J Nephrol. 2025 Jul 29;38(8):2111–27. doi: 10.1007/s40620-025-02346-x (PMC12630267; doi:10.1007/s40620-025-02346-x)
Supplement: Supplementary file 1 — Supplementary file1 (DOCX 35 KB) [file 40620_2025_2346_MOESM1_ESM.docx]

Supplemental Table 1: Search query

| **Bibliographic source** | **Search term** | **No of reports at 31/12/2024** |
| --- | --- | --- |
| Pubmed | (“arteriovenous fistula” OR “AV fistula” OR “radiocephalic fistula” OR “Cimino fistula” Or “Cimino-Brescia fistula” OR “Cimino radiocephalic fistula” OR “forearm arteriovenous fistula” OR “forearm AVF” OR “intravascular techniques” OR “percutaneous arteriovenous fistula” OR Ellipsys OR everlin Or “hemodialysis shunt” OR or "Forearm basilic*" OR "Forearm loop graft*")  AND  (“chronic kidney disease” OR “CKD” OR “chronic kidney failure” OR “end stage renal disease” OR “ESRD” OR “end stage kidney disease” OR “ESKD” OR “haemodialysis” OR “hemodialysis” OR “renal dialysis” OR “dialysis” OR “dialysis extracorporeal” OR “hemodialysis access” OR “hemodialyses” OR “renal dialyses” OR “dialyses” OR “dialyses extracorporeal” OR “hemodialyses access” OR “kidney replacement therapy”) | 51 |
| ISI - Web of science | ( "maturation rates" OR "maturation" OR "patency rates" OR "patency" OR "primary patency" OR "secondary patency" )  AND  ("radiocephalic fistula" OR "Cimino fistula" OR "Cimino-Brescia fistula" OR "Cimino radiocephalic fistula" OR "forearm arteriovenous fistula" OR "forearm AVF" OR "intravascular techniques" OR "percutaneous arteriovenous fistula" OR "Ellipsys" OR "everlin" OR "hemodialysis shunt" OR "Forearm basilic*" OR "Forearm loop graft" )  AND  ( "chronic kidney disease" OR "CKD" OR "chronic kidney failure" OR "end stage renal disease" OR "ESRD" OR "end stage kidney disease" OR "ESKD" OR "haemodialysis" OR "hemodialysis" OR "renal dialysis" OR "dialysis" OR "dialysis extracorporeal" OR "hemodialysis access" OR "hemodialyses" OR "renal dialyses" OR "dialyses" OR "dialyses extracorporeal" OR "hemodialyses access" OR "kidney replacement therapy" )) | 194 |
| Cochrane | ( "maturation rates" OR "maturation" OR "patency rates" OR "patency" OR "primary patency" OR "secondary patency" )  AND  ("radiocephalic fistula" OR "Cimino fistula" OR "Cimino-Brescia fistula" OR "Cimino radiocephalic fistula" OR "forearm arteriovenous fistula" OR "forearm AVF" OR "intravascular techniques" OR "percutaneous arteriovenous fistula" OR "Ellipsys" OR "everlin" OR "hemodialysis shunt" OR "Forearm basilic*" OR "Forearm loop graft" )  AND  ( "chronic kidney disease" OR "CKD" OR "chronic kidney failure" OR "end stage renal disease" OR "ESRD" OR "end stage kidney disease" OR "ESKD" OR "haemodialysis" OR "hemodialysis" OR "renal dialysis" OR "dialysis" OR "dialysis extracorporeal" OR "hemodialysis access" OR "hemodialyses" OR "renal dialyses" OR "dialyses" OR "dialyses extracorporeal" OR "hemodialyses access" OR "kidney replacement therapy" ) | 59 |

Supplemental Table 2: Characteristics of the studies and summary clinical data of the patients included in the systematic revision and meta-analysis

| **Author** | **Study Design** | **Continent** | **Pub. Year** | **Sample Size (patients)** | **No. Interv.** | **Mean age** | **Male (%)** | **AHT (%)** | **DLD (%)** | **DM (%)** | **SH (%)** | **CAD (%)** | **CG (%)** | **PDA (%)** |
| --- | --- | --- | --- | --- | --- | --- | --- | --- | --- | --- | --- | --- | --- | --- |
| *Wetzig et al* | Retrospective | Oceania | 1985 | 85 | 100 | 50.5 | 48.2% | N/A | N/A | N/A | 22.9% | N/A | 27.1% | 17.6% |
| *Wang et al* | Prospective | North America | 2008 | 113 | 113 | N/A | 67.3% | 77.9% | N/A | 37.2% | N/A | 31% | N/A | 0% |
| *Weale et al* | Retrospective | Europe | 2008 | 361 | 361 | N/A | 65.9% | N/A | N/A | 27.4% | 18% | N/A | N/A | N/A |
| *Zeebregts et al* | Retrospective | Europe | 2002 | 150 | 153 | 56 | 63.3% | 54% | 37.3% | 16% | 43.3% | N/A | 6.7% | 2% |
| *Prischl et al* | Retrospective | Europe | 1995 | 139 | 145 | 54.2 | 62.6% | N/A | N/A | 20.9% | N/A | N/A | 23% | N/A |
| *Alm et al* | Prospective | Europe | 1977 | 67 | 92 | 48 | 47.8% | N/A | N/A | N/A | N/A | N/A | N/A | 52.2% |
| *Lindfors et al* | Retrospective | Europe | 1976 | 45 | 45 | 42 | 66.6% | N/A | N/A | N/A | N/A | N/A | N/A | 4.4% |
| *Wong et al* | Prospective | Europe | 1996 | 60 | 60 | 58 | 61.7% | N/A | N/A | 11.7% | N/A | N/A | N/A | N/A |
| *Lin et al* | Prospective | Asia | 1998 | 176 | 176 | 57.8 | 44.9% | N/A | N/A | 40.9% | N/A | N/A | 28.4% | 0% |
| *Miller et a* | Prospective | North America | 1999 | 116 | 126 | 54 | 56.9% | N/A | N/A | 36.2% | 45.7% | N/A | N/A | N/A |
| *Golledge et al* | Prospective | Europe | 1998 | 107 | 107 | 63 | 68.2% | 75.7% | N/A | 28% | 11.2% | 37.4% | 18.7% | 0% |
| *Huseynova et al* | Retrospective | North America | 2012 | 59 | 59 | 63.9 | 74.6% | N/A | N/A | 59.3% | N/A | N/A | N/A | 0% |
| *Prasad et al* | Retrospective | Asia | 2019 | 500 | 500 | 39.9 | 82.4% | N/A | N/A | N/A | N/A | N/A | 31.8% | N/A |
| *Ramanathan et al* | Retrospective | Oceania | 2012 | 57 | 57 | 54.1 | 77.2% | 82.5% | 43.9% | 56.1% | 21.1% | N/A | N/A | N/A |
| *Anil et al* | Retrospective | Asia | 2021 | 32 | 32 | 41.5 | 81.3% | 96.9% | N/A | 31.2% | 6.2% | N/A | 25% | N/A |
| *Xu et al* | Prospective | Asia | 2023 | 124 | 124 | 54.5 | 47.6% | 71.8% | N/A | 48.4% | 39.5% | N/A | 26.6% | N/A |
| *Srivastava et al* | Prospective | Asia | 2018 | 173 | 173 | 43.3 | 69.9% | N/A | N/A | 19.1% | N/A | N/A | N/A | 4.6% |
| *Jemcov et al* | Prospective | Europe | 2013 | 122 | 122 | 55.5 | 54.1% | 98.4% | N/A | 18.9% | 46.7% | N/A | N/A | N/A |
| *O’Banion et al* | Retrospective | North America | 2015 | 61 | 61 | 57 | 77.2% | 96.7% | N/A | 78.7% | 42.6% | N/A | 1.6% | N/A |
| *Won et al* | Prospective | Asia | 2000 | 41 | 50 | 54.3 | 68.3% | N/A | N/A | 46.3% | N/A | N/A | N/A | 22% |
| *Elsayed et al* | Prospective | Asia | 2022 | 18 | 18 | N/A | N/A | N/A | N/A | N/A | N/A | N/A | N/A | N/A |
| *Bhalodia et al* | Retrospective | North America | 2011 | 58 | 58 | 59 | 74.1% | 98.3% | N/A | 56.9% | N/A | 15.5% | N/A | 27.6% |
| *Mehigan et al* | Retrospective | North America | 1982 | 154 | 154 | N/A | N/A | N/A | N/A | N/A | N/A | N/A | N/A | N/A |
| *Wolowczyk et al* | Retrospective | Europe | 2000 | 199 | 208 | 63 | 50.2% | N/A | N/A | N/A | N/A | N/A | N/A | 10.6% |
| *Bonalumi et al* | Retrospective | Europe | 1982 | 177 | 177 | 46.6 | 59.9% | N/A | N/A | N/A | N/A | N/A | N/A | N/A |
| *Twine et al* | Prospective | Europe | 2012 | 218 | 218 | N/A | 75.2% | N/A | N/A | 23.9% | 16.1% | 62.4% | 13.8% | N/A |
| *Horimi et al* | Prospective | Asia | 1996 | 139 | 139 | N/A | 81.3% | N/A | N/A | N/A | N/A | N/A | 66.2% | 0% |
| *Cassioumis et al* | Retrospective | Europe | 1992 | 173 | 173 | N/A | N/A | N/A | N/A | 11.5% | N/A | N/A | N/A | N/A |
| *Reilly et al* | Prospective | Europe | 1982 | 145 | 145 | 40.2 | 59.3% | N/A | N/A | N/A | N/A | N/A | N/A | 0% |
| *Thompson et al* | Retrospective | North America | 1972 | 77 | N/A | N/A | N/A | N/A | N/A | N/A | N/A | N/A | N/A | N/A |
| *Kherlakian et al* | Retrospective | North America | 1988 | 100 | 100 | 50 | 61% | 69% | N/A | 23% | N/A | N/A | 27% | N/A |
| *Bender et al* | Retrospective | Europe | 1994 | 56 | 56 | N/A | N/A | N/A | N/A | N/A | N/A | N/A | N/A | N/A |
| *Burt et al* | Retrospective | Europe | 2001 | 53 | 62 | 59.2 | N/A | N/A | N/A | 22.6% | N/A | N/A | N/A | N/A |
| *Lok et al* | Retrospective | North America | 2005 | 230 | N/A | N/A | N/A | N/A | N/A | N/A | N/A | N/A | N/A | N/A |
| *Mishra et al* | Prospective | Asia | 2021 | 53 | 53 | 50.9 | 91% | 60% | N/A | 70% | N/A | N/A | N/A | N/A |
| *Elshikhawoda et al* | Retrospective | Europe | 2024 | 179 | 179 | 68.7 | 57% | 69% | N/A | 70% | 50% | N/A | N/A | 17% |

Pub. Year – publication year; No. Interv. – number of interventions; AHT – arterial hypertension; DM – diabetes mellitus; DLD - dyslipidemia; SH – smoking history; CAD – coronary artery disease; CG – chronic glomerulonephritis; PDA – patient with previous dialysis access

Supplemental Table 3: Patency rates

| **Author** | **Primary Patency Rates of Fistulas at 30 days** | **Primary Patency Rates of Fistulas at 1 Year** | **Primary Patency Rates of Fistula at 2 Years** | **Primary Patency Rates of Fistulas at 3 Years** | **Secondary Patency Rates of Fistulas at 1 Year** | **Secondary Patency Rates of Fistulas at 2 Years** |
| --- | --- | --- | --- | --- | --- | --- |
| *Wong el al* | 86.54% | N/A | N/A | N/A | N/A | N/A |
| *Wetzig et al* | N/A | 76% | 73% | N/A | N/A | N/A |
| *Weale et al* | N/A | 46,4% | 32,1% | N/A | 46,4% | 32,1% |
| *Zeebregts et al* | 86.93% | 54% | 50% | 50% | N/A | N/A |
| *Prischl et al* | N/A | 48% | 39% | 36% | N/A | N/A |
| *Lin et al* | N/A | N/A | 86,4% | N/A | N/A | N/A |
| *Miller et a* | N/A | 38% | N/A | N/A | N/A | N/A |
| *Golledge et al* | 82.24% | 69% | 56% | N/A | 70% | 63% |
| *Huseynova et al* | N/A | 71% | N/A | N/A | N/A | N/A |
| *Prasad et al* | N/A | 73% | N/A | N/A | 78% | N/A |
| *Ramanathan et al* | N/A | 69,2% | 36,9% | N/A | 87.6% | 83.9% |
| *Xu et al* | N/A | 70,4% | N/A | N/A | 86.8% | N/A |
| *Bhalodia et al* | N/A | 88% | 74,1% | N/A | N/A | N/A |
| *Wolowczyk et al* | 82.21% | 65% | 58% | 58% | N/A | N/A |
| *Bonalumi et al* | N/A | 83,1% | N/A | N/A | N/A | N/A |
| *Twine et al* | N/A | 61% | 53% | N/A | N/A | N/A |
| *Horimi et al* | N/A | 96% | 89% | 87% | N/A | N/A |
| *Cassioumis et al* | N/A | 79,1% | N/A | N/A | N/A | N/A |
| *Reilly et al* | N/A | 80% | N/A | N/A | N/A | N/A |
| *Kherlakian et al* | N/A | 71% | 66% | 64% | N/A | N/A |
| *Bender et al* | N/A | 76% | 67% | 65% | N/A | N/A |
| *Burt et al* | N/A | 71,5% | 56,6% | N/A | 76,1% | 63% |
| *Lok et al* | N/A | 61,2% | 48,6% | 38,4% | N/A | N/A |
| *Mishra et al* | N/A | 81% | N/A | N/A | N/A | N/A |

NA – unavailable data

Supplemental table 4: Maturation rates and secondary variables

| **Author** | **Maturation Failure rate** | **Thrombosis rate (at 24 hours after surgery)** | **Infection rate** | **Aneurysm degeneration rates** | **Local Reintervention rate** |
| --- | --- | --- | --- | --- | --- |
| *Wetzig et al* | N/A | 13% | 0,5% | 2% | N/A |
| *Wang et al* | 37,2% | N/A | N/A | N/A | N/A |
| *Zeebregts et al* | 27,5% | 5,9% | N/A | N/A | 18,3% |
| *Prischl et al* | N/A | N/A | N/A | N/A | 38,1% |
| *Alm et al* | N/A | N/A | 6,5% | 1,1% | 27,2% |
| *Lindfors et al* | 26,7% | N/A | N/A | 8,9% | N/A |
| *Wong et al* | 20% | 3,3% | N/A | N/A | N/A |
| *Lin et al* | 13,6% | N/A | N/A | N/A | N/A |
| *Miller et a* | 25,4% | N/A | N/A | N/A | 7,9% |
| *Golledge et al* | N/A | N/A | N/A | N/A | 23,4% |
| *Huseynova et al* | 20,3% | N/A | 0,8% | N/A | 22% |
| *Prasad et al* | 16,6% | N/A | N/A | N/A | N/A |
| *Ramanathan et al* | 22,8% | N/A | 1,8% | N/A | 29,8% |
| *Srivastava et al* | 20,2% | N/A | N/A | N/A | N/A |
| *Jemcov et al* | 10,7% | N/A | N/A | N/A | N/A |
| *O’Banion et al* | N/A | N/A | N/A | N/A | N/A |
| *Won et al* | 28% | N/A | N/A | N/A | N/A |
| *Elsayed et al* | 33,3% | N/A | N/A | N/A | N/A |
| *Bhalodia et al* | 50% | N/A | N/A | N/A | N/A |
| *Mehigan et al* | 14,3% | N/A | N/A | N/A | N/A |
| *Wolowczyk et al* | 36,5% | 10,6% | N/A | N/A | N/A |
| *Bonalumi et al* | N/A | N/A | 0% | 11,3% | N/A |
| *Twine et al* | 20,2% | N/A | 20,2% | N/A | N/A |
| *Horimi et al* | N/A | 0,7% | N/A | 0,7% | N/A |
| *Reilly et al* | N/A | N/A | N/A | N/A | 73,8% |
| *Kherlakian et al* | N/A | N/A | N/A | N/A | N/A |
| *Bender et al* | N/A | N/A | N/A | N/A | 25% |
| *Burt et al* | N/A | N/A | N/A | N/A | 14,5% |
| *Elshikhawoda et al* | 37,4% | N/A | N/A | N/A | 43,6% |

NA – unavailable data

Supplemental Table 5 – Metaregression for Maturation failure

| **Variable** | **Odds Ratio (Exp(Coefficient))** | **Confidence Interval** | **p-Value** |
| --- | --- | --- | --- |
| Age | 1.007 | (1.001, 1.013) | 0.028 |
| Gender (Male) | -0.000 | (-0.001, 0.000) | 0.206 |
| Hypertension | -0.000 | (-0.001, 0.000) | 0.179 |
| DM | 1.000 | (0.999, 1.002) | 0.711 |
| Smoking | 1.002 | (0.999, 1.005) | 0.184 |
| CAD | 0.998 | (0.997, 0.999) | **< 0.001** |
| PAD | 0.994 | (0.988, 1.000) | 0.061 |
| Previous Surgery | 1.006 | (1.000, 1.011) | **0.051** |
| Cefalic Diameter | 1.076 | (0.961, 1.211) | 0.197 |
| TL | -0.000 | (-0.001, 0.000) | 0.223 |
| LL | 1.004 | (1.001, 1.008) | 0.023 |

DM – diabetes mellitus; CAD – coronary artery disease; PAD – periferic artery disease; TL (termino-lateral (End-to-Side) anastomosis); LL (latero-lateral (Side-to-Side) anastomosis)

Supplemental table 6 – Metaregression for Thrombosis

| **Variable** | **Odds Ratio (Exp(Coefficient))** | **Confidence Interval** | **p-Value** |
| --- | --- | --- | --- |
| Age | 1.003 | (0.997, 1.009) | 0.300 |
| Gender (Male) | 1.000 | (0.999, 1.000) | 0.746 |
| Hypertension | 1.000 | (0.999, 1.000) | 0.707 |
| DM | 1.001 | (0.999, 1.002) | 0.389 |
| Smoking | 1.002 | (0.999, 1.004) | 0.151 |
| CAD | 1.000 | (0.998, 1.003) | 0.749 |
| PAD | 1.004 | (0.996, 1.013) | 0.297 |
| Previous Surgery | 1.003 | (0.995, 1.010) | 0.496 |
| Cefalic Diameter | 1.081 | (0.969, 1.207) | 0.163 |
| TL | 1.000 | (1.000, 1.000) | 0.678 |
| LL | 0.999 | (0.998, 1.000) | 0.137 |

DM – diabetes mellitus; CAD – coronary artery disease; PAD – periferic artery disease; TL (termino-lateral (End-to-Side) anastomosis); LL (latero-lateral (Side-to-Side) anastomosis
